# Remnant cholesterol trajectory and subclinical arteriosclerosis: a 10-year longitudinal study of Chinese adults

**DOI:** 10.1038/s41598-024-59173-6

**Published:** 2024-04-19

**Authors:** Ping-ting Yang, Li Tang, Sai-qi Yang, Qiu-ling Shi, Ya-qin Wang, Yue-xiang Qin, Jian-gang Wang, Ying Li

**Affiliations:** 1 Department of Health Management, The Third Xiangya Hospital, Central South University, Changsha, Hunan China; 2 State Key Laboratory of Ultrasound in Medicine and Engineering, College of Biomedical Engineering, Chongqing Medical University, Chongqing, China

**Keywords:** Remnant cholesterol, Subclinical atherosclerosis, Trajectory, Longitudinal cohort, Cardiovascular disease, Cardiology, Disease prevention

## Abstract

We aimed to identify different trajectories of remnant cholesterol (RC) and investigate the association of RC trajectories with vascular endothelial function and atherosclerosis progression in a longitudinal cohort of the Chinese population. A total of 521 participants were included in the flow-mediated vasodilation (FMD) subcohort study, and 7775 participants were included in the brachial-ankle pulse wave velocity (baPWV) subcohort study. All participants had ≥ 3 medical examinations during the 10-year follow-up period. In the FMD subcohort study, three distinct RC trajectories were identified according to the RC range and changing pattern over time: “low” (57.58%), “moderate” (30.90%) and “high” (11.52%). The proportion of the three groups with vascular endothelial dysfunction (FMD < 7.0%) was 20.00%, 39.75% and 60.00% respectively. Taking the low group as a reference, participants in the moderate and high groups had over 1.88 and 2.94 times the odds of vascular endothelial dysfunction (*P* = 0.048). In the baPWV subcohort study, three distinct RC trajectories were also identified: “low” (54.29%), “moderate” (38.97%) and “high” (6.74%). The proportion of the three groups with atherosclerosis (baPWV > 1400 cm/s) was 38.79%, 51.26% and 59.01% respectively. Taking the low group as a reference, participants in the moderate and high groups had over 1.46 and 2.16 times the odds of atherosclerosis (*P* < 0.001). The findings indicated that distinct RC trajectories are significantly associated with vascular endothelial function and atherosclerosis. Regular monitoring to identify persistent increases in RC may be more helpful in identifying individuals with a high risk of cardiovascular disease.

## Introduction

Dyslipidemia is a recognized cause of cardiovascular disease (CVD), as confirmed by previous population and animal studies^1,2^. Lowering plasma low-density lipoprotein cholesterol (LDL-C) is an important approach to prevent CVD and is also included in cardiovascular disease prevention and control guidelines^3,4^. However, when LDL-C is controlled at low levels in patients, significant cardiovascular disease risk still exists^5,6^. Recently, residual cholesterol (RC), which contains chylomicron remnants, intermediate density lipoprotein and very low-density lipoprotein, has become a focus of lipid composition studies. RC has been found to be associated not only with the development of CVD but also with peripheral arterial disease, ischemic stroke, myocardial infarction, and all-cause mortality in different populations^7–12^.

The mechanisms by which RC causes various diseases are unknown, but RC has been found to induce endothelial dysfunction through oxidative and inflammatory mechanisms^13,14^. Endothelial dysfunction is considered to be an early and reversible predictor of atherosclerosis and predicts the recurrence of cardiovascular events and progression of atherosclerotic disease^15^. Atherosclerosis is the basis and mechanism of all types of vascular diseases, especially cardiovascular and cerebrovascular diseases. Clinically, flow-mediated vasodilation (FMD) measurements have been used as a method to assess endothelial function in populations^16^. The lower the FMD value is, the worse the vascular endothelial function. Brachial-ankle pulse wave velocity (baPWV) is the velocity at which a pulse wave travels along a specified artery segment and is considered an important standard for the noninvasive study of arterial stiffness^17^. The more rigid the artery is, the greater the baPWV value^18^.

Our team found an association between RC and endothelial function or atherosclerosis in a previous cross-sectional population, and higher RC was considered an independent predictor of endothelial dysfunction and atherosclerosis in participants^19^. However, due to the cross-sectional nature of the study, the single measurement data did not take into account the long-term changes and trends of RC in the population. We therefore established a longitudinal cohort with 10 years of follow-up, applying a latent class linear mixed model (LCMM) to capture all heterogeneity in individual trajectories and identify subgroups of patients with similar trajectory profiles^20,21^. This model, also known as the growth mixture model, is a type of extended standard linear mixed model for various subgroups of longitudinal trajectories. In recent years, it has been successfully used to examine the relationship between health indicator trajectories and the risk of diabetes mellitus^22,23^, cancer^24,25^and all-cause mortality^26,27^. This study proposes to identify RC trajectories and discuss their association with vascular sclerosis in a physically examined population, not only to effectively overcome the limitations of single data and better explain the causal relationship between RC and deterioration of vascular function, which may ultimately help guide lipid intervention strategies for blood lipids in the population.

## Subjects and methods

### Study design and populations

**Figure 1:**
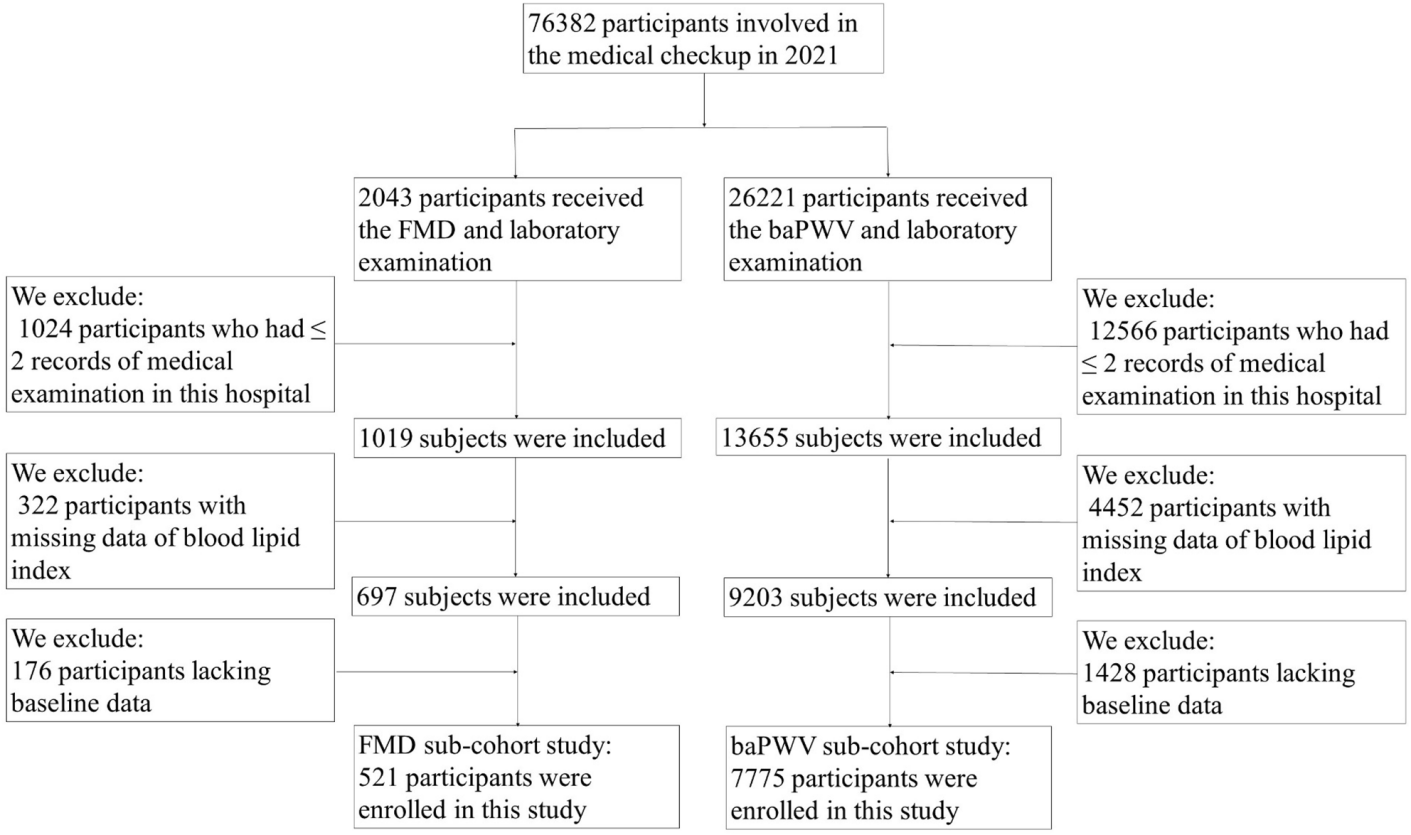
A flow diagram of participant screening and enrollment.

This cohort was built using the existing data from an ongoing longitudinal study in the Health Management Center of Third Xiangya Hospital, Hunan, China, which is one of the largest medical examination centers in China. This study cohort is dynamic and has been described in detail previously^28^. The original database was derived from the physical examination data of 1.1 million adult people, of whom 76,382 participated in the 2021 follow-up and had complete information. In this study, individuals in the FMD subcohort received FMD and laboratory tests in 2021 and completed a medical examination more than three times from January 1, 2012, to December 31, 2021 (N = 521). Individuals in the baPWV subcohort received baPWV and laboratory tests in 2021 and completed a medical examination more than three times from January 1, 2012, to December 31, 2021 (N = 7775). Each eligible participant had blood lipid data at the baseline examination (i.e., first visit) between 2012 and 2018. A flow diagram of participant screening and enrollment is shown in Fig. 1. In addition to the physical and laboratory examination, information on demographic variables (age, sex), lifestyle factors (cigarette smoking, alcohol consumption and exercise situation) and medical history were obtained by well-trained interviewers using the National Physical Examination Questionnaire^29^. Anthropometric measurements included height, weight, waist circumference (WC) and blood pressure (BP); both height and weight were measured with light clothing without shoes. Body mass index (BMI) was calculated as body weight (kg) divided by the square of body height (m). BP was measured on the right upper arm in the sitting position after a 10 – 15 min rest between 7 and 9 AM using a validated digital automatic blood pressure monitor. Informed consent was reviewed and approved by the institutional review board at the Third Xiangya Hospital (No. 2018-S389). The study was approved by an independent ethics committee at the Third Xiangya Hospital, and all the participants provided written informed consent and all methods were carried according to the general recommendations of the Declaration of Helsinki.

### FMD measurement

We evaluated the vascular response to reactive hyperemia in the brachial artery to assess endothelium-dependent FMD. High-resolution ultrasonography (UNEXEF18G, UNEX Co, Nagoya, Japan) was used to evaluate FMD. The protocol for measurement of FMD has been described in detail previously^30^. Briefly, the diameter of the brachial artery at rest was measured in the cubital region, and subsequently, the cuff was inflated to 50 mmHg above systolic blood pressure for 5 min and deflated. FMD was defined as the maximal percentage change in vessel diameter from the baseline value.

### baPWV measurement

The baPWV was measured with an automatic wave for manalyzer (BP-203 RPE III, Omron Health Medical, Dalian, China). After a minimum rest of 5 min in the supine position, 4 cuffs were wrapped around the upper arms and ankles and connected to a plethysmographic sensor (volume pulse form) and oscillometric pressure sensor. The baPWV was calculated using the formula (La − Lb)/ΔTba, in which La is the distance from the heart to the ankle, Lb is the distance from the heart to the brachium, and ΔTba is the transmission time between the brachial and posterior tibial artery waveforms. Measurements were performed twice, and the average values of the left-side and right-side assessments were calculated. Two trained technicians performed all measurements^31^.

### Biochemical parameters

Blood samples were collected according to the relevant guidelines in the Third Xiangya Hospital. All blood samples were measured using 7600 and 7170 Hitachi automatic biochemical analyzers. Fasting blood samples were collected to test fasting serum glucose (FSG), total cholesterol (TC), triglyceride (TG), high-density lipoprotein cholesterol (HDL-C) and LDL-C using LEADMAN test kits (Beijing LEADMAN Biochemical Co., Ltd. China), as well as serum creatinine (SCr) using Wako L-Type Creatinine M kits (Wako Pure Chemical Industries, Ltd. Japan). RC is defined as TC minus LDL-C minus HDL-C^32^.

### The main chronic disease definition

Hypertension was defined as self-reported hypertension diagnosed by a physician, self-reported regular use of antihypertensive medications, or systolic/diastolic blood pressure at recruitment ≥ 140/90 mmHg^33^.

Dyslipidemia was defined as meeting any of the following criteria: (1) TC ≥ 6.22 mmol/L (240 mg/dl); (2) LDL-C ≥ 4.14 mmol/L (160 mg/dl); (3) HDL-C < 1.04 mmol/L (40 mg/dl); (4) TG ≥ 2.26 mmol/L (200 mg/dl); (5) use of lipid-lowering medicines; and (6) self-reported dyslipidemia diagnosed by a physician^34^.

Diabetes mellitus was defined as self-reported diabetes diagnosed by a physician, self-reported regular use of antidiabetic medications, or FSG at recruitment ≥ 7.0 mmol/L^35^.

### Statistical analyses

**Figure 2:**
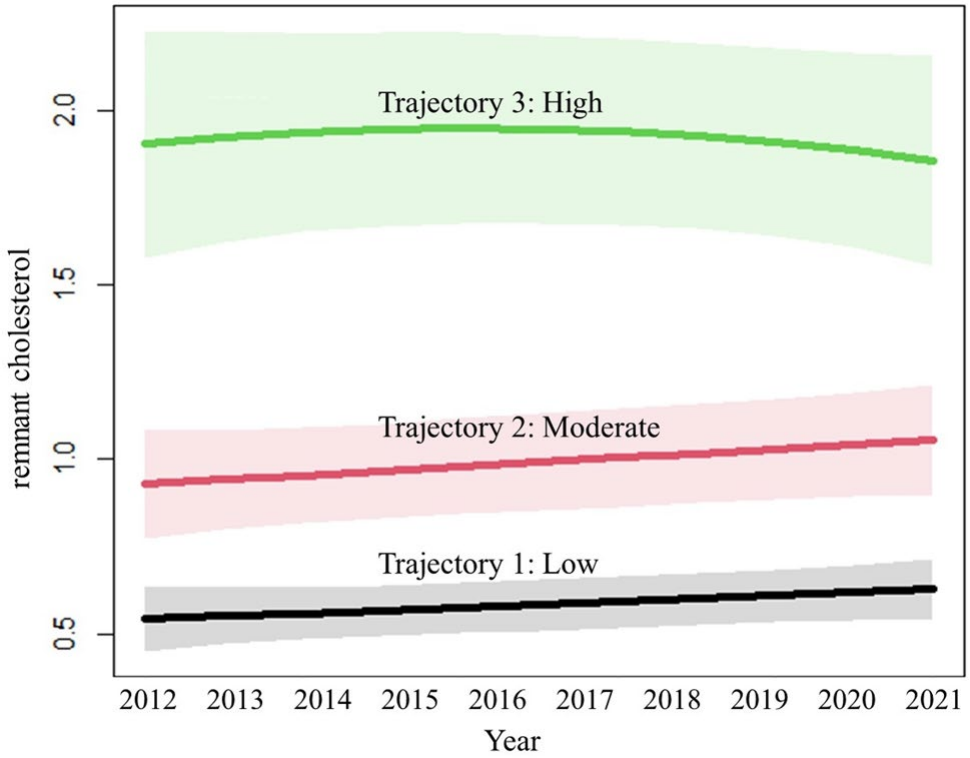
Three RC trajectories determined by the best-fit model in the FMD subcohort study from 2012 to 2021.

**Figure 3:**
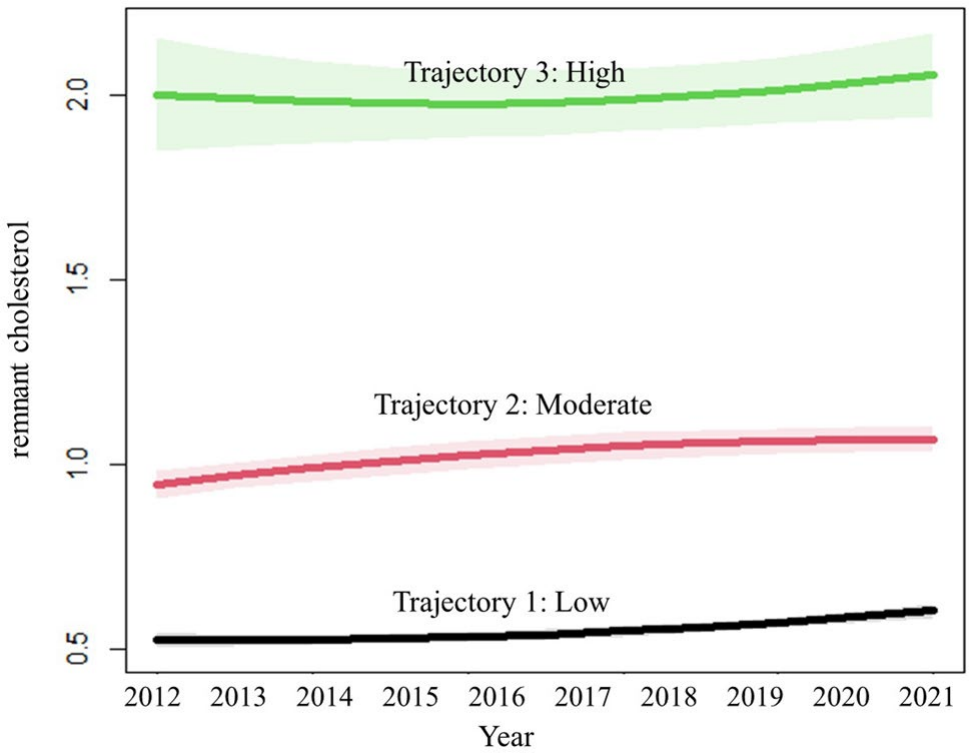
Three RC trajectories determined by the best-fit model in the baPWV subcohort study from 2012 to 2021.

Data were analyzed using Statistical Package for Social Sciences (SPSS Inc., Chicago, IL, version 22.0 for Windows) and R version 4.1.1 (R Foundation for Statistical Computing, Vienna, Austria). Continuous variables are shown as the means ± standard deviation (SD), and categorical variables are reported as percentages (%) and numbers (n). The long-term RC trajectories were identified using the latent class growth mixture modeling method. Participants with similar patterns of change in the RC from 2012 to 2021 were identified and assigned to corresponding groups. Models were fitted using the LCMM package (version 1.8.1) in R (version 3.6.1, Vienna, Austria)^20^. This method is able to handle unequally spaced or missing observations, making it possible to include participants with intermittent missing data^20^. The optimal number of clusters and adequacy of the model selected were determined using the following criteria: (1) lowest Bayesian information criterion (BIC), (2) visual inspection of graphic model curves for BIC changes, (3) average of the posterior probabilities of cluster membership for individuals assigned to each cluster exceeded 0.7, and (4) inclusion of at least 5% of participants within each trajectory cluster. In a step-by-step manner, we finally decided on the best-fitting model with three linear trajectories in both the FMD subcohort (Fig. 2) and the baPWV subcohort (Fig. 3). Comparisons of baseline characteristics by distinct RC trajectories were assessed using ANOVA with Tukey’s test for multiple groups and the χ^2^ test for categorical variables.

Previous research proposes FMD cutoff values of 4.0% and 7.0% (< 4.0% for abnormal, ≥ 4.0% and < 7.0% for borderline, ≥ 7.0% for normal) and brachial-ankle pulse wave velocity (baPWV) cutoff values of 1400 and 1800 cm/s (< 1400 for normal, ≥ 1400 and < 1800 for borderline, ≥ 1800 for abnormal)^36^. This was used as a cutoff point to determine whether there was endothelial dysfunction or arteriosclerosis. Logistic regression models were utilized to investigate the associations between RC trajectory and vascular function, with candidate variables selected using stepwise selection methods. To compare with the RC trajectories, baseline RC was divided into three groups based on RC tertiles. Potential covariates were adjusted for age, sex, BMI, WC, systolic blood pressure (SBP), diastolic blood pressure (DBP), FSG, SCr, blood urea nitrogen (BUN), uric acid (Ua), TC, TG, LDL-C, HDL-C, baseline brachial artery diameter, alcohol and smoking status, physical activity, hypertension, diabetes mellitus and dyslipidemia.

## Results

### Baseline characteristics by three trajectories of RC in the FMD subcohort study

In total, 521 participants (81.19% males) were enrolled in the study, with a median age of 43.75 years old. The baseline characteristics of the participants are presented in Table 1. The mean RC and FMD in all participants were 0.89 mmol/L and 8.81%, respectively. A total of 14.20% of the participants were diagnosed with hypertension, 42.99% with dyslipidemia and 6.71% with diabetes mellitus. In total, 53.35% of the individuals were self-reported current alcohol users, 33.01% of the individuals were self-reported current smokers and 71.78% of the individuals were self-reported current participants in sports. Participants in the low trajectory group (n = 300, 57.58%), compared to those in the moderate (n = 161, 30.90%) or high trajectory group (n = 60, 11.52%), had lower BMI, WC, DBP, SBP, FBG, SCr, Ua, TC, TG, FMD, baseline brachial artery diameter, max brachial artery diameter, current alcohol users, current smokers and prevalence of hypertension, dyslipidemia and diabetes mellitus (all *P* < 0.05). No significant difference was observed in age, sex, BUN or physical activity among the three groups. After the examination of all fitting results (Table S1), a best-fit model with three trajectories is determined and identified as low, moderate, and high. From 2012 to 2021, the low and moderate RC trajectories presented a slight elevation, while the high RC trajectory rose at first and then declined (Fig. 2). Models with 2, 4 and 5 trajectories were omitted since they were either poor in Bayesian Information Criterion or lacked subjects in the trajectory group.

**Table 1:** Baseline characteristics of FMD subcohort participants according to remnant cholesterol trajectory (mean ± SD, %N).

Characteristics	Total	Trajectory of RC			F*/x*^2^	*P*
		Low	Moderate	High		
N	521	300	161	60		
Age (years)	43.75 ± 10.49	44.18 ± 11.11	43.71 ± 9.77	41.72 ± 8.89	1.388	0.251
BMI (kg/m^2^)	24.91 ± 3.31	24.15 ± 3.22	25.79 ± 3.06	26.33 ± 3.36	20.626	< 0.001
WC (cm)	85.00 ± 9.53	82.26 ± 9.32	88.24 ± 8.53	90.00 ± 8.37	33.689	< 0.001
SBP (mmHg)	123.60 ± 14.76	122.11 ± 14.92	125.28 ± 14.47	126.59 ± 13.95	3.809	0.023
DBP (mmHg)	77.93 ± 10.89	75.88 ± 10.73	80.26 ± 10.61	82.02 ± 10.16	13.747	< 0.001
FSB (mmol/L)	5.45 ± 1.17	5.26 ± 0.84	5.63 ± 1.47	5.89 ± 1.46	10.313	< 0.001
SCr (mmol/L)	75.36 ± 14.97	73.49 ± 15.19	78.16 ± 14.41	77.23 ± 14.12	5.735	0.003
BUN (mmol/L)	4.85 ± 1.15	4.80 ± 1.09	4.95 ± 1.18	4.87 ± 1.35	0.849	0.428
Ua (mmol/L)	340.41 ± 92.90	316.06 ± 87.63	366.29 ± 91.20	392.71 ± 83.43	28.854	< 0.001
TC (mmol/L)	5.09 ± 0.97	4.95 ± 0.91	5.14 ± 1.02	5.68 ± 0.90	15.152	< 0.001
TG (mmol/L)	2.00 ± 1.78	1.22 ± 0.46	2.25 ± 0.84	5.18 ± 3.39	242.131	< 0.001
LDL-C (mmol/L)	2.78 ± 0.86	2.85 ± 0.82	2.83 ± 0.86	2.35 ± 0.93	9.001	< 0.001
HDL-C (mmol/L)	1.42 ± 0.35	1.54 ± 0.37	1.28 ± 0.24	1.16 ± 0.21	54.815	< 0.001
RC (mmol/L)	0.89 ± 0.67	0.56 ± 0.22	1.03 ± 0.39	2.16 ± 1.00	349.236	< 0.001
FMD (%)	8.81 ± 3.84	9.24 ± 3.89	8.40 ± 3.77	7.77 ± 3.51	5.034	0.007
Baseline brachial artery diameter (mm)	4.97 ± 15.61	4.16 ± 0.69	4.43 ± 0.60	10.47 ± 45.89	4.269	0.014
Max brachial artery diameter (mm)	5.37 ± 16.29	4.54 ± 0.77	4.80 ± 0.63	11.07 ± 47.94	4.208	0.015
Male % (n)	81.19 (423)	72.68 (218)	90.67 (146)	98.33 (59)	35.318	0.098
Current alcohol users % (n)	53.35 (278)	45.00 (135)	63.35 (102)	68.33 (41)	20.291	< 0.001
Current smokers % (n)	33.01 (172)	23.33 (70)	40.37 (65)	61.67 (37)	38.930	< 0.001
Physical activity % (n)	71.78 (374)	71.66 (215)	72.05 (116)	71.66 (43)	0.008	0.996
Hypertension % (n)	14.20 (74)	9.67 (29)	19.25 (31)	23.33 (14)	12.542	0.002
Dyslipidemia % (n)	42.99 (224)	21.67 (65)	62.73 (101)	96.67 (58)	151.793	< 0.001
Diabetes mellitus % (n)	6.71 (35)	4.33 (13)	8.07 (13)	15.00 (9)	9.763	0.008

*SD* standard deviation, *BMI* body mass index, *WC* waist circumference, *SBP* systolic blood pressure, *DBP* diastolic blood pressure; FSB, fasting serum glucose, *SCr* serum creatinine, *BUN* blood urea nitrogen, *UA* uric acid, *TC* total cholesterol, *TG* triglycerides, *LDL-C* low-density lipoprotein cholesterol, *HDL-C* high-density lipoprotein cholesterol, *RC* remnant cholesterol, *FMD* flow-mediated vasodilation.

### Association of RC trends with endothelial function

In this study, we first divided baseline RC into three groups according to the proportions of RC trajectories. Then, we divided baseline RC into tertiles. The association between RC and FMD reduction (cutoff value 7.0%) for different grouping methods is presented in Table 2. Among the 521 participants, 160 (30.71%) had reduced FMD in 2021. The incident rate presented an increasing trend, which was 60 (20.00%), 64 (39.75%), and 36 (60.00%) in the low, moderate, and high trajectory groups, respectively. Taking the low group as a reference, participants in the high group had approximately 3 times the odds of endothelial dysfunction (OR = 2.94; P = 0.048) after adjusting for age, sex, BMI, SBP, DBP, FSB, SCr, BUN, Ua, TC, TG, LDL-C, HDL-C, baseline brachial artery diameter, alcohol and smoking status and other factors. However, after adjusting for the above confounding factors, there was no significant correlation between the level of RC and endothelial dysfunction in the tertile group.

**Table 2:** Association of RC trajectories and categories of baseline RC with borderline FMD (cutoff values 7.0%) in FMD subcohort participants.

Variables	N	Borderline FMD (n, %)	Unadjusted	Model 1	Model 2	Model 3
			OR (95% CI)	OR (95% CI)	OR (95% CI)	OR (95% CI)
RC trajectories						
Trajectory 1: low	300	60 (20.00%)	Reference	Reference	Reference	Reference
Trajectory 2: moderate	161	64 (39.75%)	2.64 (1.73, 4.03)	2.58 (1.66, 3.99)	2.40 (1.47, 3.93)	1.88 (1.08, 3.28)
Trajectory 3: high	60	36 (60.00%)	6.00 (3.33, 10.81)	6.21 (3.35, 11.50)	5.29 (2.52, 11.16)	2.94 (1.08, 8.00)
*p* value for trend			< 0.001	< 0.001	< 0.001	0.048
Baseline RC (tertiles)						
First tertile (≤ 0.57)	174	36 (20.69%)	Reference	Reference	Reference	Reference
Second tertile (0.57 < to < 0.90)	174	43 (24.71%)	1.26 (0.76, 2.08)	1.16 (0.69, 1.95)	1.14 (0.67, 1.94)	0.88 (0.48, 1.61)
Third tertile (≥ 0.90)	173	81 (46.82%)	3.38 (2.10, 5.42)	2.91 (1.77, 4.79)	2.32 (1.23, 4.36)	1.23 (0.52, 2.93)
* p* value for trend			< 0.001	< 0.001	0.018	0.625

Model 1 was adjusted for age and sex.

Model 2 was adjusted for age, sex, alcohol and smoking status, physical activity, hypertension, diabetes mellitus, dyslipidemia, use of antihypertensive medications, use of lipid-lowering medicines and use of antidiabetic medications.

Model 3 was adjusted for age, sex, body mass index, systolic blood pressure, diastolic blood pressure, FSB, SCr, BUN, Ua, TC, TG, LDL-C, HDL-C, baseline brachial artery diameter, alcohol and smoking status, physical activity, hypertension, diabetes mellitus, dyslipidemia use of antihypertensive medications, use of lipid-lowering medicines and use of antidiabetic medications.

When FMD was less than 4.0% as the cutoff value to judge abnormal endothelial function, there was an association of RC trends with endothelial function among the 521 participants, and 31 (5.95%) had abnormal FMD in 2021 (Table 3). The incident rate presented an increasing trend, which was 8 (2.67%), 13 (8.07%), and 10 (16.67%) in the low, moderate, and high trajectory groups, respectively. However, RC grouped by trajectory (*P* = 0.293) and grouped by tertile (*P* = 0.898) were not significantly correlated with abnormal FMD after adjusting for confounding factors. The summary of baseline RC by RC trajectory groups and tertiles of baseline RC in FMD subcohort participants is shown in Table S2.

**Table 3:** Association of RC trajectories and categories of baseline RC with abnormal FMD (cutoff values 4.0%) in FMD subcohort participants.

Variables	N	abnormal FMD (n, %)	Unadjusted	Model 1	Model 2	Model 3
			OR (95% CI)	OR (95% CI)	OR (95% CI)	OR (95% CI)
RC trajectories						
Trajectory 1: low	300	8 (2.67%)	Reference	Reference	Reference	Reference
Trajectory 2: moderate	161	13 (8.07%)	3.21 (1.30, 7.91)	3.37 (1.37, 8.42)	2.96 (1.05, 8.35)	2.39 (0.73, 7.76)
Trajectory 3: high	60	10 (16.67%)	7.30 (2.75, 19.39)	8.39 (2.98, 23.59)	5.97 (1.58, 22.49)	3.58 (0.62, 20.58)
* p* value for trend			< 0.001	< 0.001	0.029	0.293
Baseline RC (tertiles)						
First tertile (≤ 0.57)	174	6 (3.45%)	Reference	Reference	Reference	Reference
Second tertile (0.57 < to < 0.90)	174	6 (3.45%)	1.00 (0.32, 3.16)	1.00 (0.31, 3.20)	0.94 (0.29, 3.06)	0.74 (2.00, 2.80)
Third tertile (≥ 0.90)	173	19 (10.98%)	3.46 (1.35, 8.88)	3.38 (1.25, 9.14)	2.08 (0.59, 7.33)	0.89 (0.17, 4.51)
* p* value for trend			0.005	0.009	0.344	0.898

Model 1 was adjusted for age and sex.

Model 2 was adjusted for age, sex, alcohol and smoking status, physical activity, hypertension, diabetes mellitus, dyslipidemia, use of antihypertensive medications, use of lipid-lowering medicines and use of antidiabetic medications.

Model 3 was adjusted for age, sex, body mass index, systolic blood pressure, diastolic blood pressure, FSB, SCr, BUN, Ua, TC, TG, LDL-C, HDL-C, baseline brachial artery diameter, alcohol and smoking status, physical activity, hypertension, diabetes mellitus, dyslipidemia use of antihypertensive medications, use of lipid-lowering medicines and use of antidiabetic medications.

### Baseline characteristics by three trajectories of RC in the baPWV subcohort study

In total, 7775 participants (67.19% males) were enrolled in the study, with a median age of 44.29 years old. The baseline characteristics of the participants are presented in Table 4. The mean RC and baPWV in all participants were 0.82 mmol/L and 1418.29 cm/s, respectively. A total of 19.78% of the participants were diagnosed with hypertension, 32.15% with dyslipidemia and 7.14% with diabetes mellitus. In total, 33.89% of the individuals were self-reported current alcohol users, 29.26% of the individuals were self-reported current smokers and 72.64% of the individuals self-reported current participation in sports. Participants in the low trajectory group (n = 4221, 54.29%), compared to those in the moderate (n = 3030, 38.97%) or high trajectory group (n = 524, 6.74%), had lower BMI, WC, SBP, DBP, FBG, SCr, BUN, Ua, TC, TG, baPWV, current alcohol users, current smokers and prevalence of hypertension, dyslipidemia and diabetes mellitus (*P* < 0.05). After the examination of all fitting results (Table S3), a best-fit model with three trajectories is determined and identified as low, moderate, and high. From 2012 to 2021, the three RC trajectories presented a slight elevation (Fig. 3). Models with 2, 4 and 5 trajectories were omitted since they were either poor in Bayesian Information Criterion or lacked subjects in the trajectory group.

**Table 4:** Baseline characteristics of baPWV subcohort participants according to remnant cholesterol trajectory (mean ± SD, %N).

Characteristics	Total	Trajectory of RC			F*/x*^2^	*P*
		Low	Moderate	High		
N	7775	4857	2269	649		
Age (years)	44.29 ± 11.51	44.22 ± 12.11	44.91 ± 10.64	42.64 ± 9.50	10.107	< 0.001
BMI (kg/m^2^)	24.44 ± 3.19	23.68 ± 3.08	25.58 ± 2.97	26.19 ± 2.83	423.523	< 0.001
WC (cm)	83.24 ± 9.72	80.58 ± 9.56	87.21 ± 8.41	89.26 ± 7.55	567.140	< 0.001
SBP (mmHg)	123.10 ± 15.37	121.11 ± 15.40	125.94 ± 15.00	128.13 ± 13.67	117.847	< 0.001
DBP (mmHg)	76.51 ± 11.15	74.61 ± 10.78	79.13 ± 11.12	81.52 ± 10.58	209.401	< 0.001
FSB (mmol/L)	5.47 ± 1.25	5.33 ± 1.07	5.59 ± 1.25	6.08 ± 2.06	122.316	< 0.001
SCr (mmol/L)	71.89 ± 16.07	69.39 ± 16.20	75.72 ± 14.96	77.24 ± 14.93	165.873	< 0.001
BUN (mmol/L)	4.70 ± 1.18	4.66 ± 1.21	4.75 ± 1.11	4.83 ± 1.19	9.044	< 0.001
Ua (mmol/L)	322.35 ± 92.44	298.91 ± 87.24	355.92 ± 87.25	380.42 ± 85.97	488.257	< 0.001
TC (mmol/L)	5.03 ± 0.94	4.83 ± 0.84	5.29 ± 0.93	5.63 ± 1.11	356.812	< 0.001
TG (mmol/L)	1.83 ± 1.64	1.14 ± 0.45	2.30 ± 0.86	5.25 ± 3.51	3777.110	< 0.001
LDL-C (mmol/L)	2.75 ± 0.83	2.73 ± 0.76	2.92 ± 0.86	2.28 ± 0.99	163.135	< 0.001
HDL-C (mmol/L)	1.47 ± 0.38	1.58 ± 0.39	1.31 ± 0.28	1.16 ± 0.25	690.083	< 0.001
RC (mmol/L)	0.82 ± 0.63	0.53 ± 0.22	1.05 ± 0.38	2.20 ± 1.09	5176.326	< 0.001
baPWV (cm/s)	1418.29 ± 300.16	1387.02 ± 301.53	1470.15 ± 292.85	1471.03 ± 282.61	71.516	< 0.001
Male % (n)	67.19 (5224)	47.16 (2762)	82.11 (1863)	92.30 (599)	649.386	< 0.001
Current alcohol users % (n)	33.89 (2635)	22.69 (1329)	41.38 (939)	56.55 (367)	297.957	< 0.001
Current smokers % (n)	29.26 (2275)	17.01 (996)	40.41 (917)	55.78 (362)	536.676	< 0.001
Physical activity % (n)	72.64 (5648)	61.31 (3591)	71.62 (1625)	66.56 (432)	17.346	< 0.001
Hypertension % (n)	19.78 (1538)	13.81 (809)	24.72 (561)	25.89 (168)	80.072	< 0.001
Dyslipidemia % (n)	32.15 (2500)	10.23 (599)	57.65 (1308)	91.37 (593)	2593.880	< 0.001
Diabetes mellitus % (n)	7.14 (555)	4.27 (250)	9.43 (214)	14.02 (91)	93.436	< 0.001

*SD* standard deviation, *BMI* body mass index, *WC* waist circumference, *SBP* systolic blood pressure, *DBP* diastolic blood pressure, *FSB* fasting serum glucose, *SCr* serum creatinine, *BUN* blood urea nitrogen, *UA* uric acid, *TC* total cholesterol, *TG* triglycerides, *LDL-C* low-density lipoprotein cholesterol, *HDL-C* high-density lipoprotein cholesterol, *RC* remnant cholesterol, *FMD* flow-mediated vasodilation.

### Association of RC trends with arteriosclerosis

We also divided baseline RC into three groups according to the proportions of RC trajectories. Then, we divided baseline RC into tertiles. The association between RC and baPWV increase (cutoff value 1400 cm/s) for different grouping methods is presented in Table 5. Among the 7775 participants, 3430 (44.12%) had increased baPWV in 2021. The incident rate presented an increasing trend, which was 1884 (38.79%), 1163 (51.26%), and 383 (59.01%) in the low, moderate, and high trajectory groups, respectively. Taking the low group as a reference, participants in the high group had 2.16 times the odds of arteriosclerosis (OR = 2.16, P < 0.001) after adjusting for age, sex, BMI, SBP, DBP, FSB, SCr, BUN, Ua, TC, TG, LDL-C, HDL-C, alcohol and smoking status, physical activity, hypertension, diabetes mellitus, dyslipidemia use of antihypertensive medications, use of lipid-lowering medicines and use of antidiabetic medications. Taking the first tertile group as a reference, participants in the third tertile group had 1.48 times the odds of arteriosclerosis (OR = 1.48, *P* < 0.001) after adjusting for the above confounding factors. When baPWV was more than 1800 cm/s as the cutoff value to judge arteriosclerosis, there was an association of RC trends with arteriosclerosis among the 7775 participants, and 807 (10.38%) had abnormal baPWV in 2021 (Table 6). The incident rate presented an increasing trend, which was 447 (9.20%), 291 (12.83%), and 69 (10.63%) in the low, moderate, and high trajectory groups, respectively. RC grouped by trajectory (*P* < 0.001 in Model 3) and grouped by tertile (*P* = 0.001 in Model 3) were both significantly correlated with abnormal baPWV after adjusting for confounding factors. The summary of baseline RC by RC trajectory groups and tertiles of baseline RC in baPWV subcohort participants is shown in Table S4.

**Table 5:** Association of RC trajectories and categories of baseline RC with borderline baPWV (cutoff values 1400 cm/s) in baPWV subcohort participants.

Variables	N	Borderline baPWV (n, %)	Unadjusted	Model 1	Model 2	Model 3
			OR (95% CI)	OR (95% CI)	OR (95% CI)	OR (95% CI)
RC trajectories						
Trajectory 1: low	4857	1884 (38.79%)	Reference	Reference	Reference	Reference
Trajectory 2: moderate	2269	1163 (51.26%)	1.66 (1.50, 1.84)	1.94 (1.60, 2.34)	1.50 (1.31, 1.72)	1.46 (1.26, 1.69)
Trajectory 3: high	649	383 (59.01%)	2.27 (1.92, 2.68)	2.43 (1.78, 3.32)	2.57 (2.05, 3.22)	2.16 (1.61, 2.91)
* p* value for trend			< 0.001	< 0.001	< 0.001	< 0.001
Baseline RC (tertiles)						
First tertile (≤ 0.57)	2513	789 (31.40%)	Reference	Reference	Reference	Reference
Second tertile (0.57 < to < 0.90)	2666	1219 (45.72%)	1.84 (1.64, 2.06)	1.45 (1.26, 1.66)	1.37 (1.20, 1.57)	1.28 (1.10, 1.50)
Third tertile (≥ 0.90)	2596	1422 (54.78%)	2.65 (2.36, 2.97)	2.16 (1.88, 2.49)	1.82 (1.53, 2.16)	1.48 (1.19, 1.84)
* p* value for trend			< 0.001	< 0.001	< 0.001	0.001

Model 1 was adjusted for age and sex.

Model 2 was adjusted for age, sex, alcohol and smoking status, physical activity, hypertension, diabetes mellitus, dyslipidemia, use of antihypertensive medications, use of lipid-lowering medicines and use of antidiabetic medications.

Model 3 was adjusted for age, sex, body mass index, systolic blood pressure, diastolic blood pressure, FSB, SCr, BUN, Ua, TC, TG, LDL-C, HDL-C, alcohol and smoking status, physical activity, hypertension, diabetes mellitus, dyslipidemia use of antihypertensive medications, use of lipid-lowering medicines and use of antidiabetic medications.

**Table 6:** Association of RC trajectories and categories of baseline RC with abnormal baPWV (cutoff value of 1800) in baPWV subcohort participants.

Variables	N	Abnormal baPWV (n, %)	Unadjusted	Model 1	Model 2	Model 3
			OR (95% CI)	OR (95% CI)	OR (95% CI)	OR (95% CI)
RC trajectories						
Trajectory 1: low	4857	447 (9.20%)	Reference	Reference	Reference	Reference
Trajectory 2: moderate	2269	291 (12.83%)	1.45 (1.24, 1.70)	1.94 (1.60, 2.34)	1.85 (1.49, 2.31)	1.96 (1.54, 2.50)
Trajectory 3: high	649	69 (10.63%)	1.17 (0.90, 1.53)	2.43 (1.78, 3.32)	2.23 (1.55, 3.21)	2.15 (1.32, 3.51)
* p* value for trend			< 0.001	< 0.001	< 0.001	< 0.001
Baseline RC (tertiles)						
First tertile (≤ 0.57)	2513	194 (7.72%)	Reference	Reference	Reference	Reference
Second tertile (0.57 < to < 0.90)	2666	284 (10.65%)	1.43 (1.18, 1.73)	1.19 (0.95, 1.50)	1.09 (0.87, 1.39)	1.17 (0.91, 1.52)
Third tertile (≥ 0.90)	2596	329 (12.67%)	1.74 (1.44, 2.10)	2.07 (1.65, 2.59)	1.78 (1.35, 2.35)	1.85 (1.31, 2.62)
* p* value for trend			< 0.001	< 0.001	< 0.001	0.001

Model 1 was adjusted for age and sex.

Model 2 was adjusted for age, sex, alcohol and smoking status, physical activity, hypertension, diabetes mellitus, dyslipidemia, use of antihypertensive medications, use of lipid-lowering medicines and use of antidiabetic medications.

Model 3 was adjusted for age, sex, body mass index, systolic blood pressure, diastolic blood pressure, FSB, SCr, BUN, Ua, TC, TG, LDL-C, HDL-C, alcohol and smoking status, physical activity, hypertension, diabetes mellitus, dyslipidemia use of antihypertensive medications, use of lipid-lowering medicines and use of antidiabetic medications.

## Discussion

In our previous research, we found a significant association between the RC and FMD as a measurement of vascular endothelial function and baPWV as a measurement of arterial stiffness in a general population^19^. However, blood lipid levels dynamically vary, and the risk of constant dyslipidemia probably contributes to the higher risk of atherosclerosis progression. Therefore, we established a longitudinal cohort study to explore the impact of RC trends on the vasculature.

During a 10-year follow-up period, 3 heterogeneous patterns of RC trajectory were identified in the FMD subcohort in this study. Taking FMD < 7.0% as the cutoff value, we found that the risk of borderline vascular endothelial dysfunction was 2.94 times higher in the high-trajectory group with the low-trajectory group as the reference after adjusting for relevant confounders. According to the grouping of trajectories, we equally divided baseline RC into three groups and found that there was no significant difference in the correlation between RC and vascular endothelial function between tertiles of different groups, independent of the baseline RC. When FMD < 4.0% was used as the cutoff value to distinguish vascular endothelial dysfunction, RC levels in either the trajectory group or the tertile group were not significantly associated with abnormal FMD, which may be related to the small sample size of 31 (5.95%) in 521 individuals who developed severe vascular endothelial dysfunction. Previous studies on RC and vascular endothelial function have focused on the fact that reducing RC can improve vascular endothelial function and mechanism studies. Hoshiga’s study found that in patients with previous coronary artery disease after 6 months of statin therapy, FMD significantly improved compared to baseline values. Changes in FMD and in total and RC cholesterol were significantly correlated^37^. Nakamura reported that patients with improved FMD showed lower percent changes in RC levels than patients who did not show improved FMD. Mediation analysis showed that the relationship between reduction in LDL-C and improvement of FMD was mediated by reduction of RC (34.5%)^38^. The mechanisms by which RC contributes to endothelial dysfunction are not fully understood. Some studies suggest that RC could impair endothelial function via direct and indirect effects on endothelial nitric oxide synthase (eNOS)^39^. Other studies have suggested that RC can enter the subendothelial space, where they are retained; within the subendothelial space, remnants induce local low-grade inflammation, endothelial dysfunction, and foam cell formation^40,41^.

In this study, there were 7775 participants in the baPWV subgroup, and three RC trajectories were fitted through heterogeneity analysis. Taking baPWV > 1400 cm/s as the cutoff value, we found that the risk of borderline arteriosclerosis was 1.48 and 2.16 times higher in the moderate-trajectory and high-trajectory groups, respectively, with the low-trajectory group as the reference, after adjusting for relevant confounders. According to the grouping of trajectories, we equally divided baseline RC into three groups and found that there was also a significant difference in the correlation between RC and borderline arteriosclerosis between tertiles. Taking the first tertile group as a reference, participants in the second and third tertile groups had 1.28 and 1.48 times the risk of borderline arteriosclerosis, respectively. Similar results were found at a cutoff point of bapwv > 1800 cm/s, implying that grouping by RC trajectory provides better discrimination than grouping by tertile of baseline RC when predicting the risk of arteriosclerosis. Recently, there were two population-based studies on the relationship between RC and baPWV. A cross-sectional study, including 912 participants from a medical health checkup center, showed that regarding lower RC as a reference, higher RC was independently associated with a higher risk of atherosclerosis, independent of other risk factors (OR = 1.794, 95% CI: 1.267–2.539, p = 0.001)^42^. Moreover, another study of 8,028 participants of a community-based atherosclerosis cohort showed that when RC was forced into the model with other lipid profile indexes simultaneously, only the RC and TG concentrations remained significantly associated with baPWV^43^. The main mechanisms by which RC contributes to the formation of atheroma plaques are as follows. Circulating plasma triglyceride-rich lipoproteins are not directly atherogenic and become deleterious only upon their conversion to smaller remnant particles. Such triglyceride-rich lipoprotein remnants are enriched in cholesterol, carrying 5–50-fold more cholesterol per particle than LDL and accounting for up to one-third of total plasma cholesterol. Cholesterol from RCs that can be retained in the subendothelial space contributes to the pathogenesis of atherosclerosis. RC induces inflammation of the arterial wall. The plasma accumulation of RC causes hyperviscosity and a procoagulant state. As a result, remnant cholesterol can be even more atherogenic than LDL-C^40,44,45^.

In this study, the trajectories of RC changes in the adult population were identified by LCMM, and it was found that the 10-year follow-up RC trajectories of the physically examined population could be classified into three groups. With the rise of RC trajectories, the long-term occurrence of endothelial dysfunction and atherosclerosis increased. Previous studies have classified trajectories for other lipid indicators but not for RC. Yu’s study identified different trajectories of lipid profiles and investigated the association of lipid trajectories with carotid atherosclerosis progression in 10,412 participants and found that borderline elevated baseline lipids (TC, TG, and LDL-C) with stable and elevated-increasing trajectories were associated with carotid atherosclerosis progression^46^. Tong’s study showed that LDL-C changing trajectories were associated with nonalcoholic fatty liver disease in men; TG and LDL-C trends were associated with an increased risk of NAFLD in women^47^. Some research found that triglyceride-glucose index trajectories are associated with an increased risk of stroke^48^, major adverse cardiovascular events in patients with diabetes mellitus^49^ and increased arterial stiffness^50^.

This study concluded that the RC trajectory approach improved risk prediction compared to a single baseline measure. The use of trajectory groupings provided better prediction of vascular dysfunction than tertile or quintile groupings. We believe that through trajectory grouping, we can better distinguish the population and identify the real high-risk population, and later intervention in this population can effectively save medical resources. To illustrate this issue, we compare the population distinguished by trajectory grouping and tertile/quintile grouping in the form of tables, as shown in Tables S5–S6. For the low-risk population, the tertile grouping had good discrimination, but for the medium- and high-risk populations, the tertile grouping had an obvious overestimation, dividing the population with lower risk levels into higher risk groups. This is not conducive to accurate management.

Our study has some limitations that should be noted. First, our data were derived from routine health checkups in a single center in China. The results of the current study may not be generalizable to other populations without these characteristics. Second, the follow-ups were unequally spaced in time, resulting in more missing follow-up data. Third, although multivariable analysis was adjusted in the logistic regression model, residual confounders were still possible, including family history, heart rate, diet, psychological, environmental and behavioral factors. Fourth, the very small number of people with FMD < 4% may account for the loss of significance and clinical meaning. Despite these limitations, this is the first study to demonstrate the association of RC trajectories with vascular endothelial function and arterial stiffness. Comprehensive analysis of the relationship between dynamic RC changes and vascular function by two subgroup cohort analyses.

## Conclusion

In conclusion, we identified that long-term high RC exposure is associated with vascular endothelial dysfunction and atherosclerosis risk. These findings indicate that regular monitoring of RC may help identify individuals with high cardiovascular disease risk. Clinicians should also pay more attention to the continuous high level of RC distribution rather than a single or occasional increase.

### Supplementary Information

Supplementary Tables.

## Data Availability

The datasets used and/or analysed during the current study available from the corresponding author on reasonable request.
