# Supplementary material for: Remnant cholesterol trajectory and subclinical arteriosclerosis: a 10-year longitudinal study of Chinese adults
Source: Sci Rep. 2024 Apr 19;14:9037. doi: 10.1038/s41598-024-59173-6 (PMC11031569; doi:10.1038/s41598-024-59173-6)
Supplement: Supplementary file 1 — Supplementary Tables. [file 41598_2024_59173_MOESM1_ESM.docx]

**Table S1. Model fit statistics by the numbers of trajectories in FMD subcohort participants.**

| Number of trajectories | Class of trajectory | Number of per class | Proportion per class | AvPP | BIC |
| --- | --- | --- | --- | --- | --- |
| 2 | 1  2 | 438  83 | 84.07%  15.93% | 0.93  0.88 | 745.15 |
| **3** | **1**  **2**  **3** | **300**  **161**  **60** | **57.58%**  **30.90%**  **11.52%** | **0.80**  **0.87**  **0.91** | **713.91** |
| 4 | 1  2  3  4 | 107  370  30  14 | 20.54%  71.02%  5.76%  2.68% | 0.82  0.73  0.83  0.92 | 734.06 |
| 5 | 1  2  3  4 | 188  72  38  207 | 36.08%  13.82%  7.29%  29.73% | 0.85  0.74  0.84  0.99  0.87 | 741.0533 |
|  | 5 | 16 | 3.08% |  |  |

AvPP, average posterior possibility; BIC, Bayesian information criterion; AvPP >0.7 indicates good classification accuracy.

Lower absolute values of BIC indicate better fitness.

**Table S2. Summary of baseline RC by RC trajectory groups and tertiles of baseline RC in FMD subcohort participants.**

|  | **Mean ± SD** | **Maximum value** | **Minimum value** |
| --- | --- | --- | --- |
| **RC trajectories** (mmol/L) |  |  |  |
| Trajectory 1: low | 0.56 ± 0.22 | 1.66 | 0.06 |
| Trajectory 2: moderate | 1.03 ± 0.39 | 3.36 | 0.31 |
| Trajectory 3: high | 2.16 ± 0.99 | 4.89 | 0.63 |
| **Baseline RC (tertiles)** (mmol/L) |  |  |  |
| First tertile | 0.41 ± 0.11 | 0.57 | 0.06 |
| Second tertile | 0.71 ± 0.09 | 0.90 | 0.58 |
| Third tertile | 4.89 ± 0.78 | 4.89 | 0.90 |

**Table S3. Model fit statistics by the numbers of trajectories in baPWV subcohort participants.**

| Number of trajectories | Class of trajectory | Number of per class | Proportion per class | AvPP | BIC |
| --- | --- | --- | --- | --- | --- |
| 2 | 1  2 | 6649  1126 | 85.52%  14.48% | 0.93  0.89 | 9590.233 |
| **3** | **1**  **2**  **3** | **4221**  **3030**  **524** | **54.29**%  **38.97**%  **6.74**% | 0.81  0.85  0.91 | 8396.865 |
| 4 | 1  2  3  4 | 3186  2487  1732  370 | 40.38%  31.29%  22.38%  4.75% | 0.73  0.73  0.81  0.92 | 8129.634 |
| 5 | 1  2  3  4 | 250  1682  2816  2264 | \| 3.22% \| \| --- \| \| 21.63% \| \| 36.22% \| \| 29.12% \| | 0.92  0.69  0.69  0.72  0.75 | 8062.951 |
|  | 5 | 763 | 9.81% |  |  |

AvPP, average posterior possibility; BIC, Bayesian information criterion; AvPP >0.7 indicates good classification accuracy.

Lower absolute values of BIC indicate better fitness.

**Table S4. Summary of baseline RC by RC trajectory groups and tertiles of baseline RC in baPWV subcohort participants.**

|  | **Mean ± SD** | **Maximum value** | **Minimum value** |
| --- | --- | --- | --- |
| **RC trajectories** (mmol/L) |  |  |  |
| Trajectory 1: low | 0.53 ± 0.22 | 1.61 | 0.06 |
| Trajectory 2: moderate | 1.05 ± 0.38 | 2.91 | 0.25 |
| Trajectory 3: high | 2.20 ± 1.09 | 5.85 | 0.21 |
| **Baseline RC (tertiles)** (mmol/L) |  |  |  |
| First tertile | 0.36 ± 0.08 | 0.49 | 0.06 |
| Second tertile | 0.65 ± 1.00 | 0.84 | 0.50 |
| Third tertile | 1.44 ± 0.74 | 5.85 | 0.85 |

**Table S5. Differences between trajectory groupings and tertile groupings in the FMD subcohort**

|  | Total | Trajectory 1: Low | Trajectory 2: Moderate | Trajectory 3: High |
| --- | --- | --- | --- | --- |
| Frist tertile (≤0.57) | 174 | 162(93.10%) | 12(6.90%) | 0(0%) |
| Second tertile (0.57＜to＜0.90) | 174 | 121(69.54%) | 51(29.31%) | 2(1.15%) |
| Third tertile (≥0.90) | 173 | 17(9.83%) | 98(56.65%) | 58(33.52%) |

**Table S6. Differences between trajectory groupings and quintile groupings in the FMD subcohort**

|  | Total | Trajectory 1: Low | Trajectory 2: Moderate | Trajectory 3: High |
| --- | --- | --- | --- | --- |
| Frist quintile (≤0.44) | 105 | 101 (96.19%) | 4 (3.81%) | 0 (0%) |
| Second quintile (0.44＜to≤0.62) | 104 | 89 (85.58%) | 15 (14.42%) | 0 (0%) |
| Third quintile(0.62＜to≤0.79) | 104 | 72 (69.23%) | 31 (29.81%) | 1 (0.96%) |
| Fourth quintile(0.79＜to≤1.18) | 104 | 33 (31.73%) | 63 (60.58%) | 8 (7.69%) |
| Fifth quintile (>1.18) | 104 | 5 (4.81%) | 48 (46.15%) | 51 (49.04%) |
